# An effective video inpainting technique using morphological Haar wavelet transform with krill herd based criminisi algorithm

**DOI:** 10.1038/s41598-024-66496-x

**Published:** 2024-07-05

**Authors:** M. Nuthal Srinivasan, M. Chinnadurai, S. Senthilkumar, E. Dinesh

**Affiliations:** 1 Department of Electronics and Communication Engineering, E.G.S. Pillay Engineering College, Nagapattinam, Tamil Nadu 611002 India; 2 Department of Computer Science and Engineering, E.G.S. Pillay Engineering College, Nagapattinam, Tamil Nadu 611002 India; 3 Department of Electronics and Communication Engineering, M. Kumarasamy College of Engineering, Karur, Tamil Nadu 639113 India

**Keywords:** Video inpainting, Criminisi algorithm, Krill herd optimization, Down sampling, Wavelet decomposition, Haar wavelet, Electrical and electronic engineering, Computer science

## Abstract

In recent times, video inpainting techniques have intended to fill the missing areas or gaps in a video by utilizing known pixels. The variety in brightness or difference of the patches causes the state-of-the-art video inpainting techniques to exhibit high computation complexity and create seams in the target areas. To resolve these issues, this paper introduces a novel video inpainting technique that employs the Morphological Haar Wavelet Transform combined with the Krill Herd based Criminisi algorithm (MHWT-KHCA) to address the challenges of high computational demand and visible seam artifacts in current inpainting practices. The proposed MHWT-KHCA algorithm strategically reduces computation times and enhances the seamlessness of the inpainting process in videos. Through a series of experiments, the technique is validated against standard metrics such as peak signal-to-noise ratio (PSNR) and structural similarity index (SSIM), where it demonstrates superior performance compared to existing methods. Additionally, the paper outlines potential real-world applications ranging from video restoration to real-time surveillance enhancement, highlighting the technique’s versatility and effectiveness. Future research directions include optimizing the algorithm for diverse video formats and integrating machine learning models to advance its capabilities further.

## Introduction

Video inpainting is a weighty method for achieving content restoration^1^. It is mainly utilized for recovering visualized content by removing subtitles, logos, superimposed stamps, novel tickers on frames, or enhances the video quality which has endured specific alterations. A practical application of inpainting involves restoring old videos containing valuable information that have deteriorated due to natural disasters, aging, or poor storage conditions. While it is easy to identify areas that need restoration, the process of digitally repairing these damaged regions is complex and challenging. Another commercial scheme is for removing unnecessary data from video frames. Several earlier television platforms have implanted with subtitles, or everyday news ticker closer to the frame boundary. Currently, these programs are illustrated or broadcasted somewhere worldwide, these expired subtitles or text with distinct languages should be removed earlier to the injecting the target data. The video inpainting is applied to eliminate the actual subtitle or text in an invisible manner, thus public could adore the video without being attentive to modification. Additionally, this method has been utilized with data embedded in the data hidden application.

In video inpainting, the design of individual image inpainting is considered for several frames or images treating in a video^2^. The individual image inpainting uses the present data from the whole damaged regions for rehabilitation. Conventional image inpainting techniques deliberated in a relevant survey are categorized to the texture synthesis method and Partial Differential Equation (PDE) image inpainting techniques^3^. PDE-based technique mainly estimates the gradient vector from nearby inpainting areas. Besides, the lines are vertical to neighboring linear structure and gradient vectors are dispersed in the pixels regions that need inpainting. These methods are well suitable to restore bigger regions; however, it tends to execute poor images with minimum repetition textures. Conventional video inpainting techniques use patch-based optimization approaches for filling lost areas with sample patches from known areas. This technique frequently suffered from restricted vulnerability and efficiency toward complicated movements. The innovative operation in digital inpainting^4^ applies nonlinear PDE as an interpolation framework for performing video and image frame inpainting. The ides of interpolation and PDE in inpainting are utilized in several methods, involving that derive third order PDE depending upon Taylor expansion for propagating the boundary isopods to the lost areas. A specific growth of this method presented in^5^ is introduced in^6^ that employ Navier-Stoke formulation. This method employs the concept from traditional fluid dynamics for continuous propagating isophote lines of the image from external inpainting area. Additional method, the presented video inpainting system in^7^ benefit from separate p-Laplacian regularization on a weight graph. In spite of their possible outcomes, the interpolation and PDE based approaches execute frame by frame completion which ignores the continuousness over successive frames until PDEs are adjusted in a three-dimension system^8^. Furthermore, this technique is suitable for small and narrow lost areas.

Video inpainting has seen substantial advancements through the integration of wavelet transforms and machine learning models, reflecting its growing importance in both academic and practical domains. Recent developments, such as in^9^ illustrate the innovative use of wavelet transformations combined with transformer networks to address challenges in video inpainting. This approach particularly emphasizes the enhancement of temporal consistency in video streams, a crucial aspect for maintaining narrative continuity in restored footage.

Similarly,^10^ highlights the versatility of wavelet methods beyond traditional video, extending into medical imaging. This work reviews various wavelet-based techniques for denoising computed tomography (CT) images, highlighting the method's efficacy in reducing noise while preserving important clinical details. Moreover,^11^ demonstrates the application of wavelet methods in satellite and radar image processing. This research addresses the despeckling of synthetic aperture radar (SAR) images, enhancing the quality of experience in real-time remote sensing, crucial for environmental monitoring and disaster response. Lastly, the^12–15^ integrates wavelet analysis with deep learning architectures to specifically target noise-robust inpainting. This hybrid approach utilizes the strengths of both technologies to improve the reliability and quality of video restoration, particularly in scenarios affected by significant noise and disruption. These diverse applications and developments not only demonstrate the breadth of video inpainting research but also set the stage for our proposed technique, which aims to further advance the field by integrating Morphological Haar Wavelet Transform with Krill Herd based Criminisi algorithm (MHWT-KHCA). Our work builds on these foundational studies to address specific challenges in video inpainting, such as high computational costs and the need for maintaining high-quality output in resource-constrained environments.

This paper presents a new Video Inpainting Technique using Morphological Haar Wavelet Transform with Krill Herd based Criminisi algorithm, called MHWT-KHCA algorithm. The presented MHWT-KHCA algorithm performs inpainting in different stages such as frame extraction with down sampling, decomposition, and inpainting. The presented model involves MHWT technique-based decomposition process, which generates a low-resolution image with set of sub-bands of wavelets. Besides, KHCA technique is utilized on the sub bands for inpainting the missing regions. Particularly, the KHCA technique is derived to eliminate the high computational complexity of traditional CA, and the KHCA algorithm results in effective searching of the optimal matching block in the unbroken regions. For evaluating the efficacy of the MHWT-KHCA technique, a set of experiments were executed and the outcomes are examined under several measures.

## Literature review

In recent times, Convolutional Neural Network (CNN) was initially utilized in^16^ for image inpainting; however, it only for smaller holes. Pathak et al.^17^ presented for handling the huge lost area by an encoder decoder framework that could effectively study the background features of the image. For image inpainting with high resolution, a multi scale neural patch synthesis technique is introduced that not only maintains background construction but also generates higher details^18^. The technique presented in^19^ additionally enhances the efficiency by including 2 adversary losses for measuring the local and global reliability of the outcomes. Distinct from the past works that only concentrates on box shaped hole, it also establishes an approach for handling the holes with random shape. Extending this technique from image to video field is a difficult process, due to video completion, it is not only required for having a precise context knowledge of both motions and frames but also need to guarantee temporary softness of the output. This method presented a new deep learning (DL) framework for this issue that utilizes both two and three-dimensional CNNs to combine for learning the spatial and temporal structures in detail^20^.

An additional sequence of processes by utilizing three-dimension CNN for three-dimension structure accomplishment is provided. Related to DL based image inpainting, all techniques are utilized encoder decoder framework however three-dimension CNN is used for resolving this challenge. But, most of the methods could only manage low resolution grids (typically 303 voxels) because of higher computation cost of three-dimension convolution. In order to resolve this problem, several methods have recently been presented. Dai et al.^21^ utilized patch assembly and retrieval as a post-processing for refining the low-resolution outcome of encoder-decoder system. For this post refinement, the techniques in^22^ presented an approach for slicing the low-resolution outcome to a series of images also performed completion and super resolution for every sliced image with a repeated NN. A hybrid network is implemented to joint universal structure forecast and local geometry interpretation^23^. This research work is stimulated by these techniques however it varies from it in 2 features. Initially, the technique accompanied the completion in an area growing manner whereas it utilizes end to end framework for conclusion. Next, for detailed inference, the technique is only looking at a local region that lacking nearby context data whereas this technique utilizes the content of the entire image for lost data retrieval.

In recent times, various DL based methods are separated into two types. Initially, it mostly consists of Short- and Long-Term Context Aggregation Net for Video Inpainting five, which is based on short term reference data if inpainting targeted the frame. For instance, VINet^24^ utilizes a repeated encoder-decoder system for collecting data in nearby frames by flow warping based background accumulation. A multi-stage architecture is presented for video inpainting: it is initially utilizing deep flow completion network for restoring the flow order, later execute backward and forward pixel propagation by utilizing the returned flow order, and at last, it utilizes a pre-trained image inpainting method for refining the outcomes^25^. The secondary model utilizes a suitable group of frames in the whole video as reference data. Wang et al.^26^ presented a 2-stage method with integration of 2D and 3D CNNs. CPNet^27^ proposed a context aggregation by forecasting affine matrices and employing affine transformation over fixed sample reference frames.

A simple fusion based inpainting model fuses the color and depth features to overcome the modality gaps in inpainting process^28^. The proposed guided color and depth inpainting network attained better inpainting results compared to traditional methods. The self-attention model presented in^29^ provides a global to local progressive network for video inpainting. The inpainting approach has local and global self-attention model to enhance the search efficiency and accuracy. Due to this, local redundancy caused due to textures are reduced in the inpainting process and provides better reliability.

A fast Fourier convolution-based video inpainting model presented in^30^ incorporates transformer networks to enhance the inpainting performances. The presented model initially represents a token considering the frame wide and then the token is fed into spatio-temporal transformer to predict the missing areas in large scale. Better contextual information can be obtained using the transformer network. However, the memory requirements of transformer networks are high and it requires large training data and computationally intensive compared to recent models. Similar transformer-based video inpainting model presented in^31^ utilizes deep encoder and an attention block to attain better performances. The features from the video are effectively obtained through deep encoder and the incorporated attention block recreates the attributes and merge together to provide a complete spatial structure. However, the deep encoder has limited contextual understanding as it lags to understand the long-term dependencies of video frames.

The discrete wavelet transforms based video inpainting model presented in^32^ process the low and high frequency components in video frames through different feature aggregation models to generate the missing features. Finally using a gradient weighted network, the reconstruction is performed with better reconstruction results. Similar wavelet-based transformer presented in^33^ considers the spatial information while performing video inpainting. In addition to spatial and temporal learning procedure, the presented model includes an attention model which considers low frequency and high frequency band losses while reconstructing the images. However, handling diverse textures and motion patterns require additional training procedure which limits the presented approach performances.

An automatic video inpainting is proposed in the literature to focus on dynamic textures construction, moving of multiple objects, and moving background^34^. In fact, achieving high-quality inpainting results on high-definition movies has only lately been feasible, and even then, only partially automatically. An automatic approach utilising mid-level structural clues to guide patch-based image completion was presented^35^. This proposed technology struggled to provides good results for high quality video and image inpainting.

## The proposed MHWT-KHCA algorithm

The working principle of the proposed MHWT-KHCA technique is given here. The MHWT-KHCA technique follows a three-stage procedure, namely frame extraction with down sampling, decomposition, and inpainting. Firstly, the input videos which need to be inpainted are given as input to the MHWT-KHCA technique. Then, the frames in the videos are extracted and down sampled to get low-resolution images. Followed by, the decomposition of the frames takes place using MHWT technique and creates a sub-band of wavelets of a low-resolution image. Finally, the KHCA technique is applied to the subbands for inpainting the missing regions.

### Frame extraction and downsampling

In the MHWT-KHCA video inpainting process, the selection of the target region, or the area to be inpainted, is a crucial step that precedes the actual inpainting action^36^. This selection is typically automated and based on a detection algorithm that identifies missing or damaged areas in the video frames. The process involves several key steps:

1. The video frame is initially analyzed to detect anomalies or disruptions in visual continuity using edge detection and texture analysis techniques. This helps in identifying areas that lack coherent visual data compared to their surroundings.
2. Once potential target areas are identified, they are marked as regions of interest (ROI). This marking is based on a thresholding method where pixels significantly differing from their surrounding in terms of color, intensity, or texture are flagged.
3. The marked regions are then validated to ensure they indeed represent missing or corrupted data. This validation might involve temporal consistency checks across successive frames to confirm the persistence of detected anomalies.
4. After validation, these areas are defined as target regions for inpainting. The boundaries of these regions are refined using morphological operations to ensure smooth transitions for the inpainting process.
5. Within the target regions, priority is assigned to different areas based on the algorithmic needs. Factors such as the proximity to high-detail areas, the structural importance of the region within the frame, and the extent of damage or data loss can influence priority levels

### Frame extraction and downsampling

Once the target regions within the video frames are identified and validated, the next step involves the precise extraction and preparation of these frames to facilitate effective inpainting. This transition is not only critical for maintaining the quality and consistency of the video but also sets the stage for applying sophisticated downsampling techniques that optimize the processing load. At the beginning stage, frame extraction from video takes place and the downsampling process is achieved by a factor of 2 for getting images with low resolution. Since processing each high dimension frame individually will increase the computation complexity. Thus, the frames are down sampled so that the spatial resolution is reduced and smaller sized images will be obtained. Moreover, down sampling ensures that neighboring frames in the video have similar spatial characteristics, so that a better temporal consistency can be obtained during the inpainting process. Inpainting missing regions across successive frames with better temporal consistency will provide smoother and more visually appealing results. The low-resolution image inpainting is independent of noise which means noise may be less prominent in low-resolution images compared to high-resolution images due to the reduced level of detail^37,38^. However, the proposed model incorporated bilateral filter to avoid the boundary noises and preserves the edges in the video frames. Once the low-resolution images are attained, the present image gets subtracted from the earlier image. When the residual becomes 0 or less than the threshold pixel value, there is no need for inpainting process is indicating any difference between the present and previous frames. This process eliminates the inpainting of the repetitive frames. When the residual is not equivalent to 0, the inpainting procedure is performed.

### MHWT based decomposition process

At this stage, the MHWT technique is used to decompose video frames into subbands of wavelets. The primary purpose of choosing MHWT-based decomposition is to break down the input video frame into various frequency bands, achieving a multiscale representation. This multiscale representation can provide different levels of features, allowing for effective extraction of local and global structures in the video frames. Moreover, MHWT can retain spatial localization information in the decomposition process, which provides a significant advantage for inpainting. Unlike traditional wavelet transforms, which suffer from blurring effects, MHWT can preserve the edges and boundary information of objects in the video frames. Compared to recent deep learning models that rely on large amounts of annotated data for training, MHWT doesn’t require large datasets and is computationally efficient. Thus, for a balanced multiscale representation, edge preservation, spatial localization information, and computational efficiency, MHWT is proposed for the feature extraction process.

Mathematically, the Haar transform matrix is defined using Eq. (1):

1 $$T=HF{H}^{T}$$

where $F$ is a $N\times N$ and $H$ denotes the $N\times N$ image and transformation matrices, $T$ is the outcome using the Haar transform. $H$ Comprises the Haar basis function ${h}_{k}\left(z\right)$, that can be represented using an unbroken locked interval of $z\in [0, 1],$ $k=0, 1, 2, N-1$, where $N={2}^{n}$. For creating the $\text{H}$ matrix, the integer variable $k$ can be defined by $k={2}^{p}+q-1$ ($0\le p\le n-1$ , if $=0$ , $q=0$ or 1; if $p\ne 0$, $1\le q\le {2}^{p}$). The Haar basis function is denoted by

2 $${h}_{0}\left(z\right)={h}_{00}\left(z\right)=\frac{1}{\sqrt{N}}, z\in \left[0, 1\right]$$

and

3 $${h}_{k}\left(z\right)={h}_{pq}\left(z\right)=\frac{1}{\sqrt{N}}\left\{\begin{array}{l}{2}^{p/2}\left(q-1\right)/{2}^{p}\le z<\left(q-0.5\right)/{2}^{p}\\ {2}^{p/2}\left(q-0.5\right)/{2}^{p}\le z<q/{2}^{p}\\ 0\text{ others},z\in \left[\text{0,1}\right]\end{array}\right.$$

The $ith$ row of $N\times N$ Haar transform matrix includes the components hi $\left(z\right)$, where $z=0/N,$ $1/N,$ $2/N,$ $\left(N-1\right)/N.$

Then, the morphological version of Haar wavelet is developed by substituting the linear signal analysis filter of Haar wavelet with the erosion (or dilation), for instance, considering the lower (or higher) over two instances.

Assume a collection of ${V}_{j}$ of signal spaces^39^ where $\text{j}$ might exist over a finite or infinite index set. The signal analysis operator ${\psi }_{j}^{a}\left(x\right): {V}_{j}\to {V}_{j+1}$ maps ${V}_{j}$ into ${V}_{j+1}$ and the detailed analysis operator ${\omega }_{j}^{a}\left(x\right):{V}_{j}\to {W}_{j+1}$ maps ${V}_{j}$ into ${W}_{j+1}$. The family ${\psi }_{j}^{S}$ of signal synthesis operator and ${\omega }_{j}^{S}$ of detail synthesis operators maps ${V}_{j+1}$ back into ${V}_{j}$. It is represented as:

4 $${\psi }_{j}^{a}\left(x)(e,f\right)=\text{min}\left(x\left(2\text{e}, 2f\right),x\left(2\text{e}+1, 2f\right),x\left(2e+1, 2f\right),x\left(2\text{e}+1, 2f+1\right)\right)$$

5 $${\omega }_{j}^{a}\left(x\right)\left(e,f\right)=\left({\omega }_{j,v}^{a}\left(x\right)\left(e,f\right), {\omega }_{j,h}^{a}\left(x\right)\left(e,f\right), {\omega }_{j,d}^{a}\left(x\right)\left(e,f\right)\right)$$

where ${\psi }_{j}^{a},$ ${\omega }_{j, v}^{a},$ ${\omega }_{j,h}^{a},$ ${\omega }_{j, d}^{a}$ denoted the scaled signal and the vertical, horizontal, and diagonal detail signals, that are represented as

6 $$ \omega _{{j,v}}^{a} (x)(e,f) = 1/2(\left( {x\left( {2e,2f} \right) - x\left( {2e,2f + 1} \right) + x\left( {2f + 1,2f} \right) - x\left( {2f + 1,2f + 1} \right)} \right) $$

7 $${\omega }_{j, h}^{a}\left(x\right)\left(e,f\right)=1/2\left(\left(x(2e, 2f\right)-x\left(2e,+1, 2f\right)+\text{1,2}f\right)+\text{x}(2e,2f+1)-x(2e+1, 2f+1))$$

8 $$ \omega _{{j,d}}^{a} {\text{ }}x\left( {e,f} \right) = 1/2\left( {\left( {x)(2e,2f} \right) - x(2e + 1,2f} \right) - x\left( {2e,2f + 1} \right) + x\left( {2e + 1,2f + 1} \right)) $$

The above vertical, horizontal, and diagonal components can provide the multiscale texture and structural information in the video frame. In the inpainting process performed in the next stage, these features are used to know the missing region in the video frame. By ensuring the nearby textures and structures the missing regions can be painted effectively.

The synthesis operators are provided by

9 $${\psi }_{j}^{\text{s}}\left(x\right)\left(2e, 2f\right)={\psi }_{j}^{s}\left(x)(2e,2f+1\right)={\psi }_{j}^{s}\left(x\right)\left(2e+\text{1,2}f\right)={\psi }_{j}^{\text{s}}\left(x\right)\left(2e+1, 2\text{f}+1\right)=x\left(e,f\right)$$

And

10 $${\omega }_{j}^{s}\left(\text{y}\right)\left(2\text{e}, 2\text{f}\right) =\text{m}ax\left({y}_{v}\left(e,f\right)\right)+{y}_{h}(e,f),{y}_{\mathcal{V}}(e,f)+{y}_{d}(e,f),{y}_{h}(e,f)+{y}_{d}\left(e,f\right),0)$$

11 $${\omega }_{j}^{s}\left(\text{y}\right)\left(2\text{e}+1, 2\text{f}\right)=\text{m}ax\left({y}_{v}\left(e,f\right)\right)+{y}_{h}(e,f),{y}_{\mathcal{V}}(e,f)+{y}_{d}(e,f),{y}_{h}(e,f)+{y}_{d}\left(e,f\right),0)$$

12 $${\omega }_{j}^{s}\left(\text{y}\right)\left(2\text{e}, 2\text{f}+1\right) =\text{max}({y}_{h}\left(e,f\right)-{y}_{v}\left(e,f\right),{-y}_{\mathcal{V}}\left(e,f\right){-y}_{\mathcal{V}}\left(e,f\right) +{y}_{d}(e,f),{y}_{h}(e,f)+{y}_{d}\left(e,f\right),0)$$

13 $${\omega }_{j}^{s}\left(\text{y}\right)\left(2\text{e}+1, 2\text{f}+1\right)=\text{max}({-y}_{v}\left(e,f\right)-{y}_{h}\left(e,f\right),{y}_{d}\left(e,f\right){-y}_{\mathcal{V}}\left(e,f\right),{y}_{d}\left(e,f\right),-{y}_{h}\left(e,f\right),0)$$

where we write $y\in {\omega }_{j}^{S}$ as $y=\left(yv, yh, yd\right)$ .

### KHCA based video inpainting process

Finally, the KHCA based video inpainting process takes place to fill the omitted regions in the input image. The omitted regions in the input image are detected using an automated anomaly detection mechanism that identifies discrepancies in continuity and texture. This is accomplished by analyzing the frequency and spatial information derived from the MHWT decomposed frames. Areas lacking coherence with surrounding pixels are flagged as omitted and prioritized for inpainting by the KHCA. This detection is critical for accurately defining the target areas where the inpainting algorithm needs to focus, ensuring a seamless restoration of the video. The KHCA is derived by incorporating the characteristics of KH algorithm with the classical Criminisi algorithm and it effectively searches the optimal matching block in the unbroken regions. Criminisi model derives an efficient technique of integrating the merits of PDE and texture synthesis dependent inpainting^40–42^. A summary of the operation involved in the Criminisi technique is discussed here. Consider an input image as 1, the target image is represented by $\Omega$ and contour $\delta \Omega ,$ $\Phi =l-\Omega$ is the source area and target and source patches can be defined as ${\Psi }_{p}$ and ${\Phi }_{q}$ respectively. At present, a standard mask size of 9 $\times$ 9 pixels can be used as the patch region. The steps involved in the Criminisi method are defined as follows.

**Figure 1:**
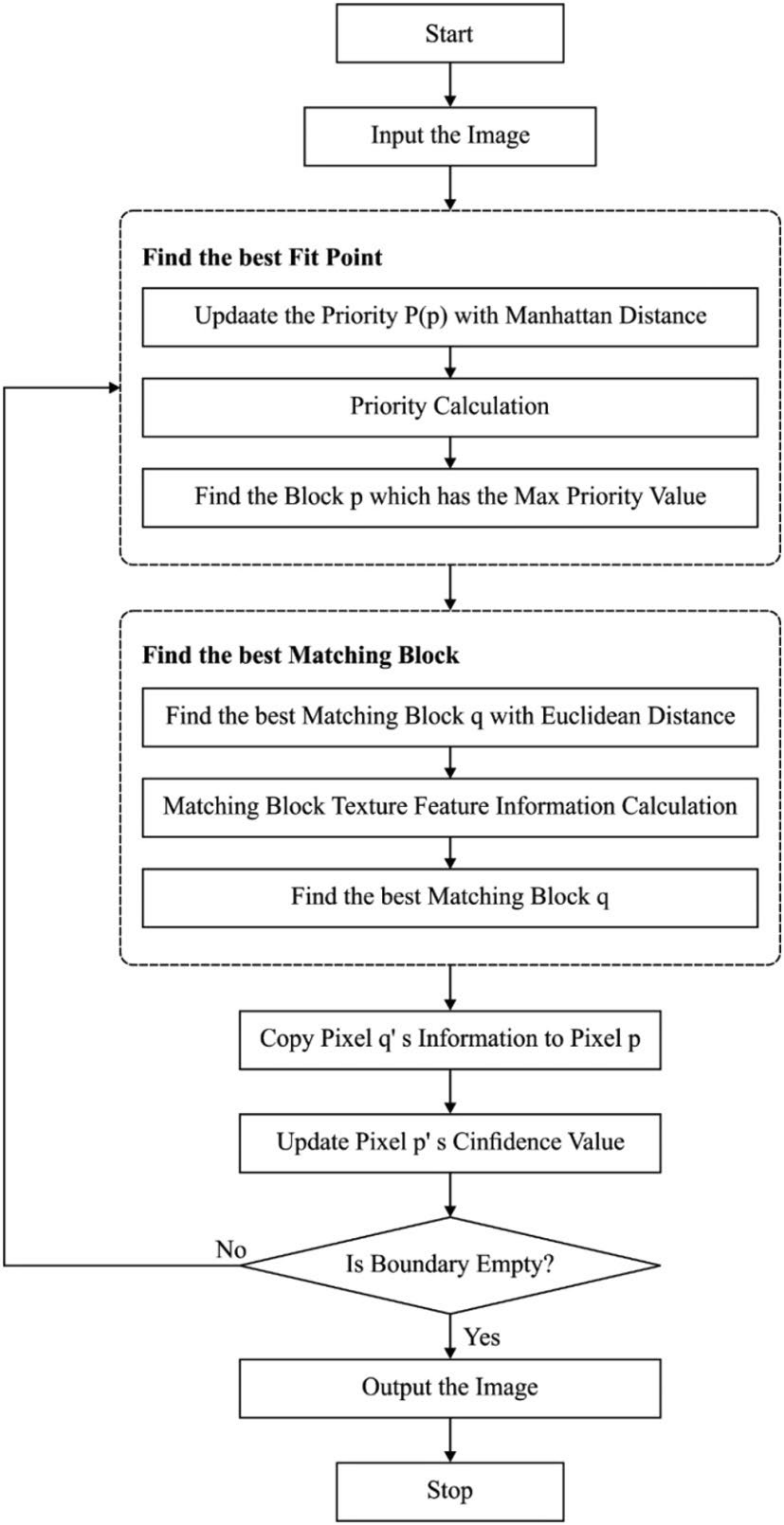
Flowchart of Criminisi algorithm.

- **Step 1.** Choose the target region $\Omega$ of the image and the remaining portion of the image are considered as source region $\Phi .$
- **Step 2.** To identified the contour $\delta \Omega$ of target region $\Omega ,$ ${\text{if}}_{c\Omega }=\varnothing ,$ stop; besides, carry out the following steps.
- **Step 3.** Every patch ${\Psi }_{p}$ positioned at the point $\left(\text{p}\in \delta \Omega \right)$, calculate the priority $P\left(p\right)$ using the multiplication of 2 elements:
  14 $$P\left(\text{p}\right)=C\left(\text{p}\right)\times D\left(\text{p}\right)$$
  The confidence $C\left(p\right)$ determines the number of trustworthy data from the patch ${\Psi }_{p}$ that can be equated as:
  15 $$C\left(\text{p}\right)=\frac{{\Sigma }_{q\in ({\Psi }_{p}\cap \Phi \rangle B\left(\text{p}\right) }}{|{\Psi }_{p}|} ,$$
  where $|{\Psi }_{p}|$ indicates the count of pixels that exists from the patch, ${\Psi }_{p}$ , $B\left(p\right)$ signifies the region of identified parts in the patch ${\Psi }_{p}$. The data term $D\left(p\right)$ implies the strength function of isophote hits the contour $\delta \Omega$ all iterations and determined by:
  16 $$D\left(\text{p}\right)=\frac{|\nabla {l}_{P}^{\perp }*{n}_{p}|}{\alpha },$$
  where $\alpha$ is the normalization factor and ${n}_{p}$ represents the unit normal vector that is orthogonal to present contour $\delta \Omega$ at $p$, and $\nabla {l}_{p}^{\perp }$ designates direction and intensity of iso-illuminance line of point $p.$
- **Step 4.** Next to the identification of the patch ${\Psi }_{p}$ with maximum priority, the technique starts to search for an optimal matching patch ${\Phi }_{\text{q}}$ in the source regions. A patch ${\Phi }_{\text{q}}$ requires to fulfil^32^:
  17 $$d={\text{arg}}_{\text{q}\in \Phi }\text{min}d\left({\Psi }_{\widehat{p}},{\Phi }_{\text{q}}\right) ,$$
  where distance $d\left({\Psi }_{\widehat{p}}, {\Phi }_{\widehat{q}}\right)$ determines the sum of squared difference (SSD) of the identified pixel amongst the patches ${\Psi }_{p}$ and ${\Phi }_{\text{q}}$ is represented as:
  18 $$d\left({\Psi }_{\widehat{p}},{\Phi }_{\widehat{p}}\right)={\sum }_{i=1}^{N}{\left({C}_{pi}-{C}_{\text{q}i}\right)}^{2}$$
  For gray scale image, $C$ means image, and $N$ specifies the count of pixels where ${\Psi }_{p}$ is placed in $\Phi$. In case of color images,
  19 $$d\left({\Psi }_{\widehat{p}}{\Phi }_{\widehat{p}}\right)={\sum }_{\text{i}=\text{l}}^{N}\left[{\left({R}_{pi}, -{R}_{\text{qi}},\right)}^{2}+{\left({G}_{pi}-{G}_{\text{qi}}\right)}^{2}+{\left({B}_{pi}-{B}_{\text{q}i}\right)}^{2}\right]$$
- **Step 5.** Duplicate the recognized pixel of the patch ${\Phi }_{\widehat{q}}$ to the equal unidentified pixel of patch ${\Psi }_{\widehat{p}}$ and update the confidence value. At last, the above processes get iterated still the missing pixel is entirely filled. Figure 1 demonstrates the flowchart of Criminisi Algorithm^43,44^.

The CA suffers from high computational complexity. Upon finding the optimal matching block, it searches the entire image apart from the missing regions. In the original CA, this priority order is typically determined based on the confidence and coherence of neighboring pixels. However, these heuristics may not always lead to globally optimal solutions, especially in complex inpainting scenarios. Moreover, it tries to offer maximum searching accurateness which increases the computational time. For resolving this issue, KH algorithm is applied in the proposed work. By integrating Krill Herd optimization, the process of selecting the priority order can be enhanced. KH can explore different solutions (i.e., priority orders) in the solution space and adaptively adjust the priority order based on the collective behavior of the swarm. Due to this more efficient exploration of the solution space can be obtained and improves the overall inpainting performances.

KH^45^ is a novel metaheuristic optimization technique applied to resolve optimization process that is dependent upon the swarm of krills regarding specific environmental and biological processes. Krill is one of the best studied species and its main characteristics is its ability to form large swarms. However, when a krill is attacked by a predator, the swarm removes the individual krill which reduces the krill density. After predation the krill is again formed with objective to increase the krill density, and reach the optimal food source. Thus, the optimization model considers the distance between food location and increasing the krill density in the objective function to solve the global optimization problems. An individual krill moves towards the best solution while search for the food and krill density. The optimization process is explained through three major steps as follows.

i. Motion influenced by another krill individuals,
ii. Forage actions,
iii. Arbitrary diffusion.

#### Initialization

Better search space abilities are essential for an optimization model. In KH optimization, the algorithm should have the capability to search for the solution in arbitrary dimensions. Thus, a Lagrangian model is generalized to an $d$‐dimensional decision and presented as follows

20 $$\frac{d{X}_{i}}{d\text{t}}={N}_{i}+{F}_{i}+{D}_{i},$$

where ${N}_{\text{i}},$ ${F}_{\text{i}}$, and ${D}_{\text{i}}$ signifies movement guided by other krill individuals, physical diffusion of the ith krill individual, and the foraging movement, correspondingly.

#### Motion influenced by another krill individuals

Generally, the krill individuals try to main the herd density as high and perform movement considering the mutual effects of other krill. When the motion subjective by another krill individuals, the route of motion, ${\alpha }_{\text{i}}$, is nearly calculated by local outcome (local swarm density), repulsive result, and target outcome. Thus, for a krill individual, this motion is determined by

21 $${N}_{i}^{\text{new}}={N}^{\text{ max }}{\alpha }_{\text{i}}+{\omega }_{n}{N}_{i}^{\text{old}}$$

where ${N}^{\text{ max}}$ denotes maximal induce speed, ${\omega }_{n}$ indicates inertia weight of the movement induce in $[0, 1]$, and ${N}_{\text{i}}^{\text{old}}$ represents final movement induced. The local effect provided by the neighbors $\left({\alpha }_{i}^{local}\right)$ and target direction effect provided by best individual $\left({\alpha }_{i}^{target}\right)$ are collectively presented as ${\alpha }_{\text{i}}$ which is mathematically expressed as

22 $${\alpha }_{\text{i}}={\alpha }_{i}^{local}+{\alpha }_{i}^{target}$$

where

23 $${\alpha }_{i}^{local}=\sum_{j=1}^{{N}_{n}}{\widehat{k}}_{i,j}{\widehat{x}}_{i,j}$$

24 $${\widehat{k}}_{i,j}=\frac{{k}_{i}-{k}_{j}}{{k}^{worst}-{k}^{best}}$$

25 $${\widehat{x}}_{i,j}=\frac{{x}_{i}-{x}_{j}}{\Vert {x}_{i}-{x}_{j}\Vert +\varepsilon }$$

where the krill individual worst and best fitness are represented using ${k}^{worst}$ and ${k}^{best}$. The fitness and position of individual krill is represented using $k$ and $x$. The number of neighbors is indicated using ${N}_{n}$. a small positive value $\varepsilon$ is added to avoid singularities. To select the neighbors the optimization model calculates the neighborhood ratio so that the number of closest krill individuals can be identified. The sensing distance is determined for all the krill individual which is mathematically expressed as

26 $${d}_{s}=\frac{1}{lN}\sum_{j=1}^{N}\Vert {x}_{i}-{x}_{j}\Vert $$

where sensing distance is indicated using ${d}_{s}$ and krill individual is indicated using $N$. $l$ is a numerical factor which is in the range of^1,5^. Considering sensing distance, if the distance is less the krill are considered as neighbors. Further the effect of individual krill with best fitness is considered and formulated as

27 $${\alpha }_{i}^{target}={c}^{best}{\widehat{k}}_{i,best}{\widehat{x}}_{i,best}$$

where ${c}^{best}$ indicates the coefficient of individual with best fitness and it is expressed as

28 $${c}^{best}=2\left(rand+\frac{I}{{I}_{max}}\right)$$

where $I$ indicates the iteration and ${I}_{max}$ indicates the maximum iteration and $rand$ indicates the random value and its range is given as [0,1].

#### Forage actions

**Figure 2:**
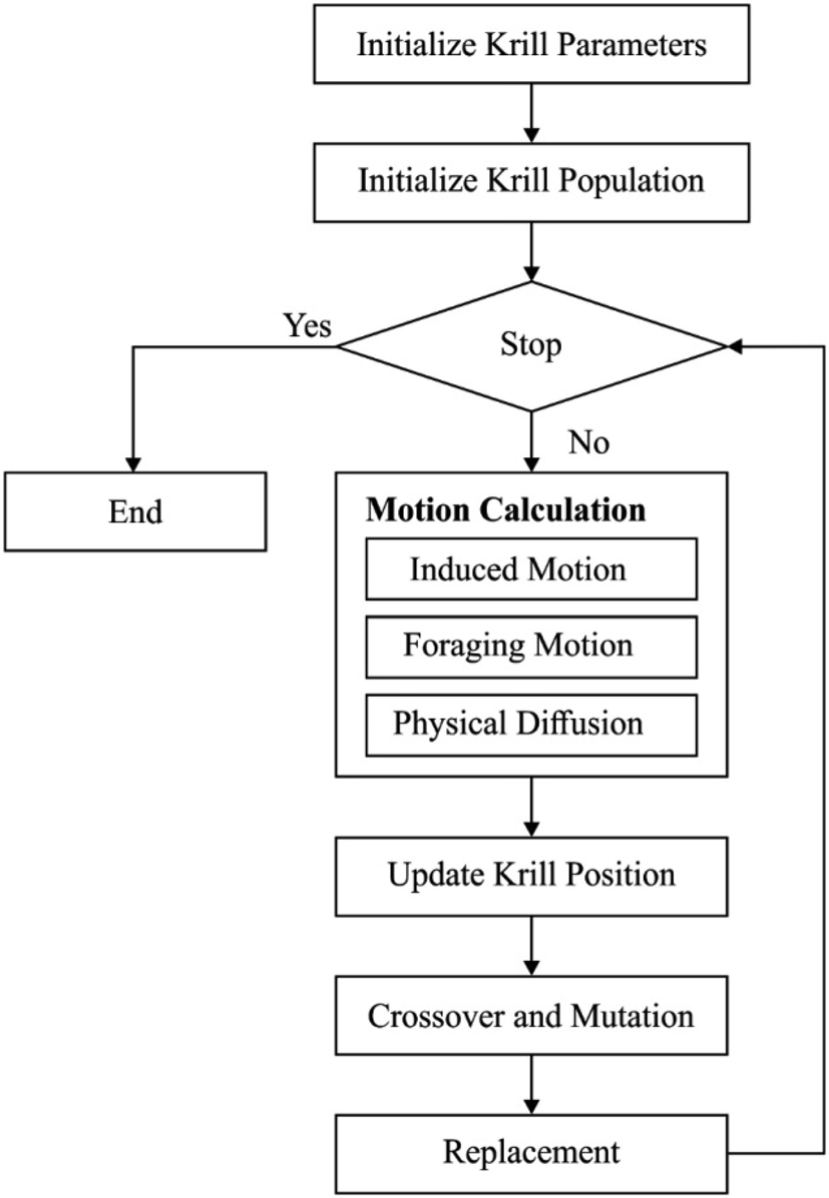
Flowchart of KH algorithm.

The foraging movement is calculated by 2 major elements. Initially, it represents food position and next, it denotes previous knowledge with the food spot. For ith krill individual, this movement is nearly connected as:

29 $${F}_{\text{i}}={V}_{f}{\beta }_{\text{i}}+{\omega }_{f}{F}_{\text{i}}^{\text{old}},$$

where

30 $${\beta }_{i}={\beta }_{i}^{\text{food}}+{\beta }_{i}^{\text{best}},$$

and ${V}_{f}$ denotes foraging speed, ${\omega }_{f}$ represents inertia weight of foraging movement among zero and one, ${F}_{\text{i}}^{\text{old}}$ indicates final foraging movement. The flowchart of KH technique is shown in Fig. 2.

#### Arbitrary diffusion

The arbitrary function is assumed to be arbitrary diffusion of krill individual. This movement is defined with respect to a maximal diffusion speed and arbitrary direction vector. It is represented as:

31 $${D}_{\text{i}}={D}^{\text{ max }}\delta ,$$

where ${D}^{\text{ max}}$ denotes maximal diffusion speed, and $\delta$ represents arbitrary direction vector and their arrays are arbitrary values from − 1 and 1.

According to 3 above mentioned motions, by distinct variables of the movement in the time, the position vector of krill individual in the interval $t$ to $t+\Delta t$ is stated as:

32 $${X}_{i}\left(\text{t}+\Delta \text{t}\right)={X}_{i}\left(\text{t}\right)+\Delta t\frac{d{X}_{i}}{d\text{t}}.$$

It needs to be renowned that $\Delta t$ is the major significant variable and fine-tuned with respect to particular real-time engineering optimization problems. Due to this variable, it is denoted as scale factor of the speed vector. Additional information regarding the 3 major movements and KH technique is made in^36,37^.

At the CA, the error square sum algorithm (SSD) through low computation time is commonly employed as a matching condition. So, the SSD is employed for measuring the island suitability index as defined below:

33 $$SSD\left(i,j\right)=\frac{1}{M\times N}\sum_{s=1}^{M}\sum_{t=1}^{N}{\left[S\left(i+s-1,j+t-1\right)-T\left(s,t\right)\right]}^{2},$$

where $S$ denotes the pixel value of pixels $s(i+s-1, j+t-1)$ from the block is occupied, and $T$ represents the pixel value of point (s, t) from the optimal matching block. $M, N$ denotes the horizontal as well as vertical coordinate points of pixels correspondingly. The lower value of error matching results in lower SSD and maximum island adaptability index. Thus, the optimal solution of KH optimization model refines the selection process of CA to select features from the neighbor regions. Also, the exploration and adaptive procedures of KH provides more choices to CA to select appropriate features over time to ensure better inpainting performance. Thus, CA dynamically update the priority order so that this can be used in film scenes so that better patch choices can be provide while ensuring the motion consistency.

## Performance validation

**Figure 3:**
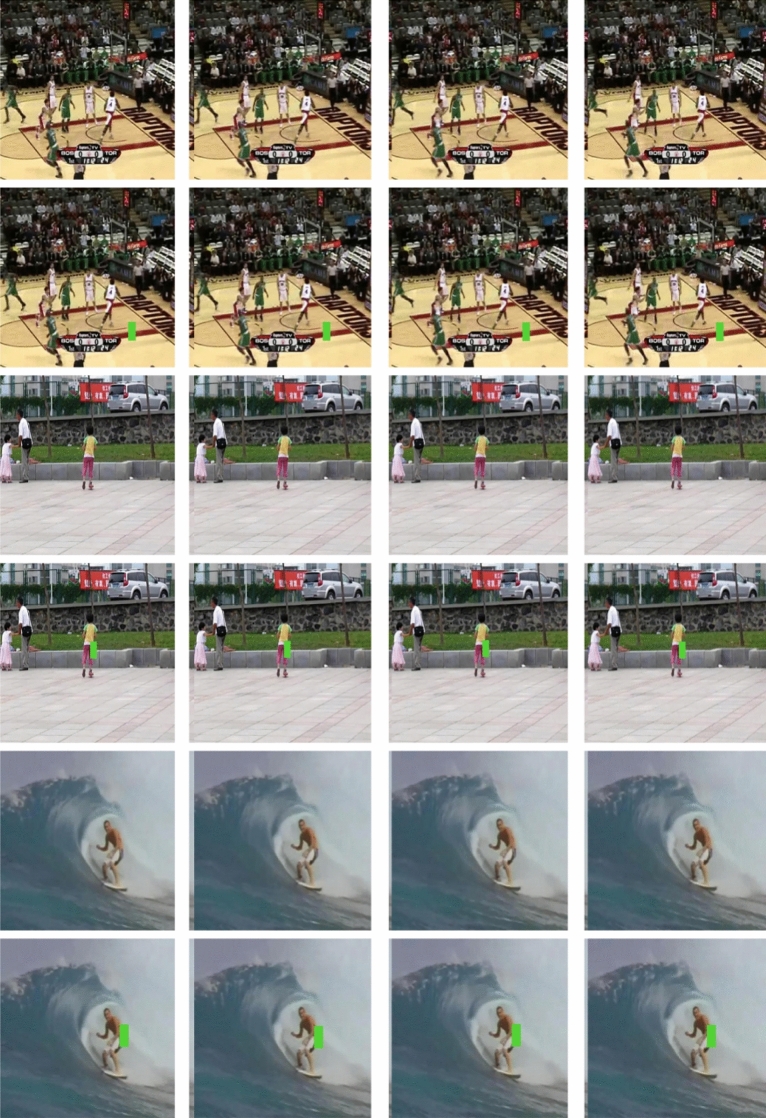
Input: Second row/fourth row/sixth row and Output: first row/third row/fifth row.

In this section, performance analysis for validation of the proposed systems is presented. The experimentation of proposed model utilizes benchmark OTB2015 dataset^46^ from Kaggle repository. The dataset has 100 commonly used video sequences for visual tracking. The dataset has different videos like basketball playing video, biker, bird, car, boy, girl, surfing, skating, etc., The video resolution is 1280 × 720 pixels with frame rate of 30fps. In the proposed model experimentation after extracting the frames, down sampling is performed by a factor of 2 to get 640 × 360 sized frames. The experimentations are done in MATLAB 2019 and the performance metrics like PNSR, SSIM, and Computation time are evaluated. The outputs obtained using the proposed model is presented in Fig. 3. Figure illustrates the visualization results obtained by the presented model on the applied input videos with missing region. From the figure, it is clear that the 2nd, 4th, and 6th rows demonstrate the input video frames with missing regions, and the corresponding inpainting output videos with filled regions are exhibited in the 1st, 3rd, and 5th rows. It is apparent that the presented model correctly fills the missing regions.

**Figure 4:**
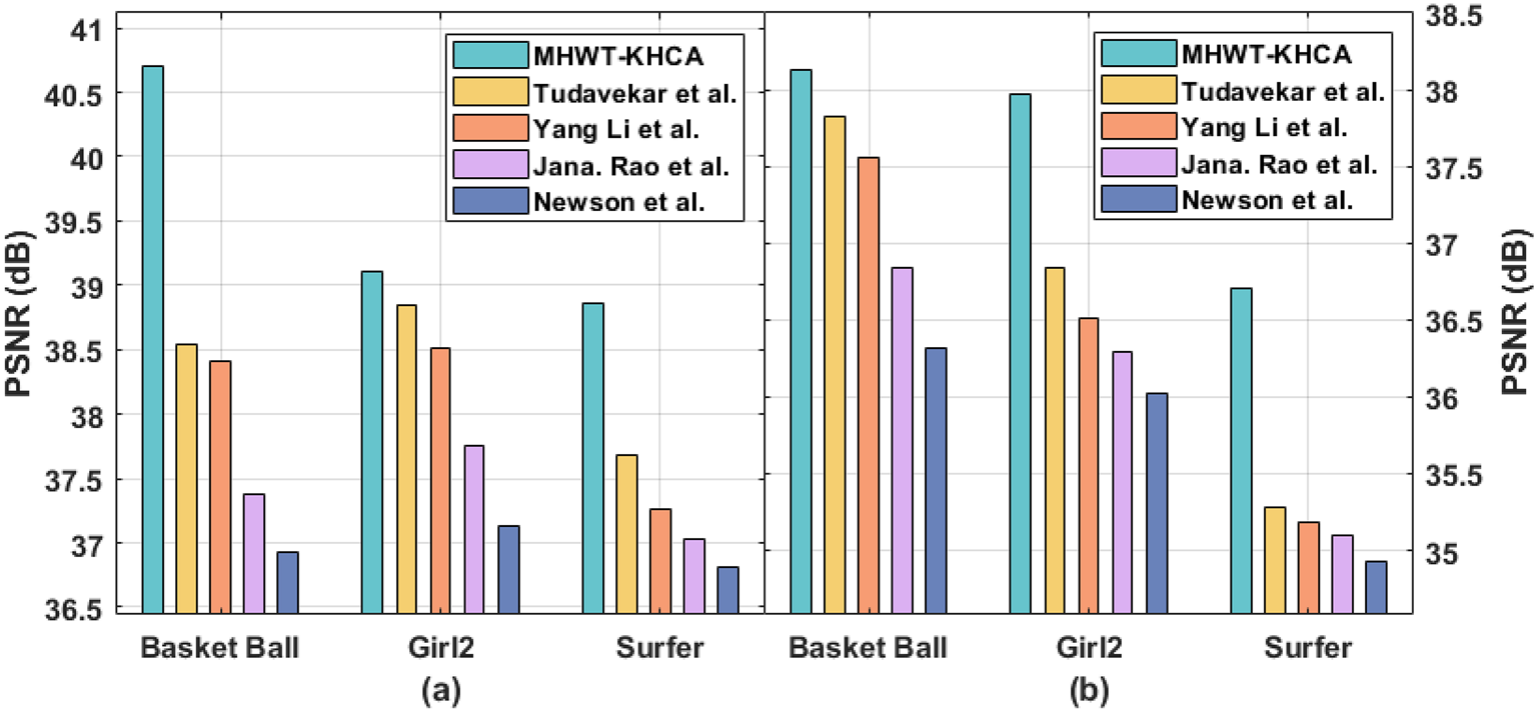
PSNR (**a**) Error level 15% (**b**) Error level 30%.

**Figure 5:**
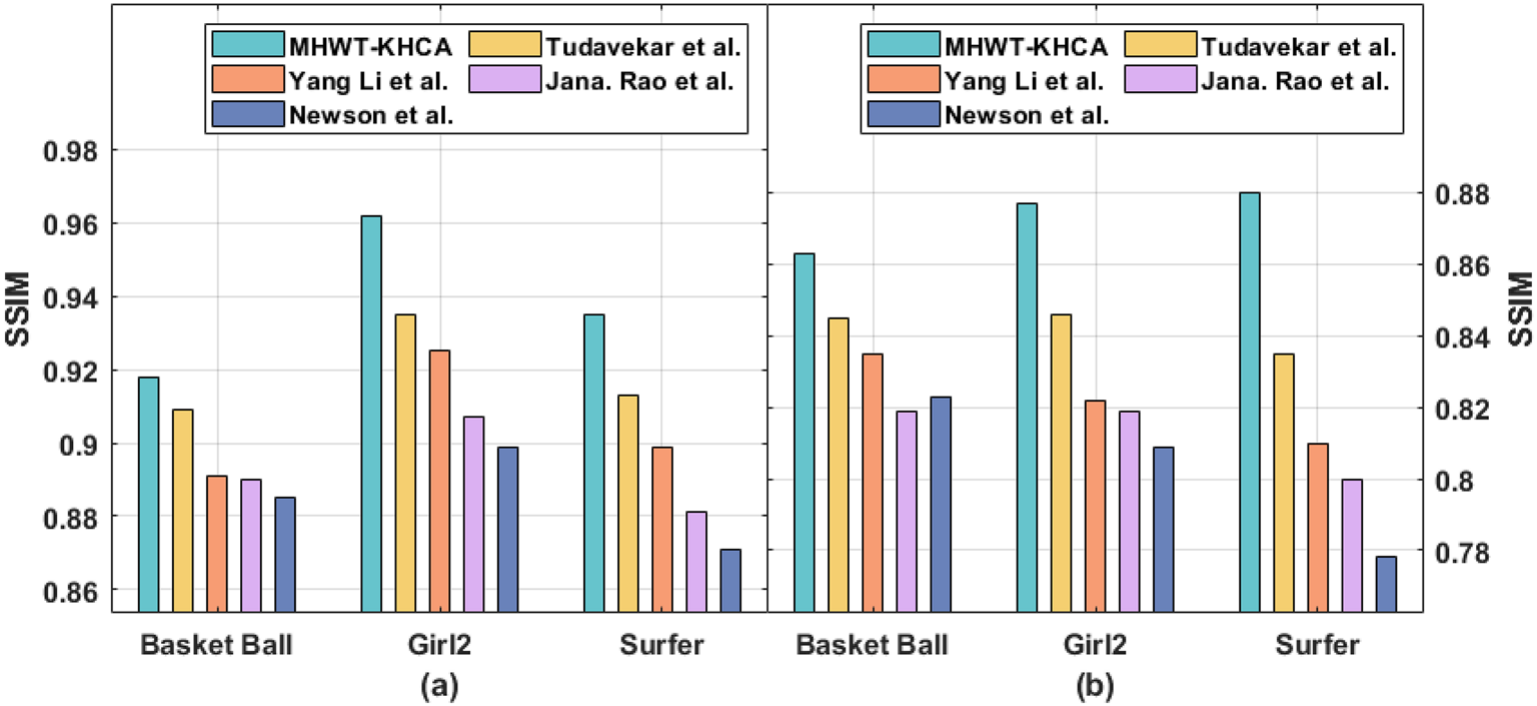
SSIM (**a**) Error level 15% (**b**) Error level 30%.

**Figure 6:**
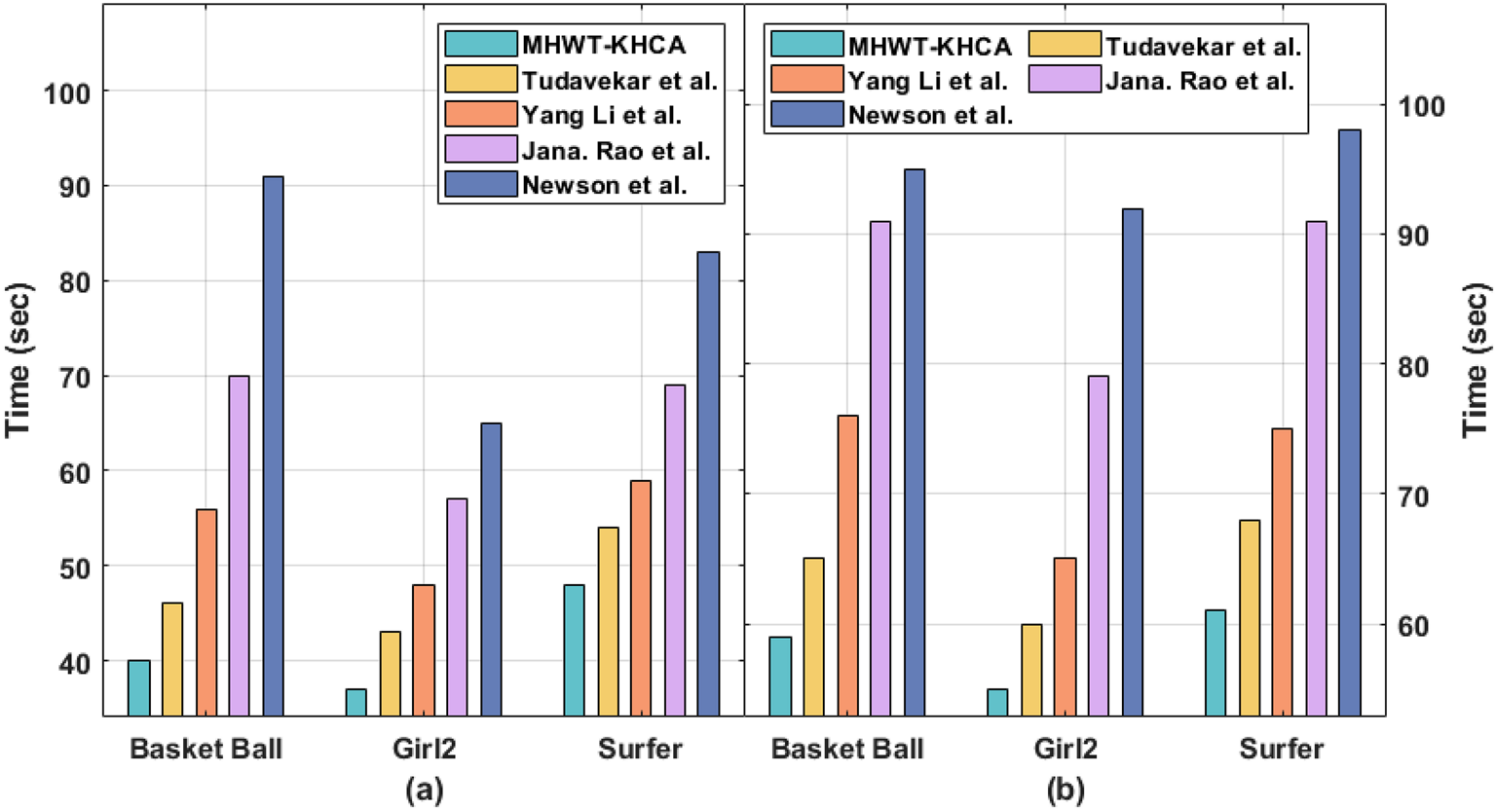
Time (**a**) Error level 15% (**b**) Error level 30%.

To validate the proposed model better performance with already established research works in this same domain, we have considered research works of Tudavekar et al., Yang Li et al., Jana. Rao et al., and Newson et al. The existing methods utilized the same dataset and evaluated the performance metrics which same as in the proposed model. Table 1 and Figs. 4, 5, 6 demonstrates the comparative results analysis of the MHWT-KHCA model with other existing methods in terms of PNSR, SSIM, and CT. Specifically the proposed model superior performance is highlighted with minimum execution time when the pixel loss is at 15% and 30%. The issue identified in the Yang Li et al. research work that utilizes an encoder and decoder network is it takes more than a minute to fill missing pixels in a 512 × 512 image. Similarly, the fuzzy based inpainting model of Tudavekar et al. takes nearly a minute to fill the missing pixels.

**Table 1:** Result analysis of existing with proposed model in terms of various performance measures.

Video Seq	Pixel loss (%)	Methods	PSNR	SSIM	Time (s)
Basket ball	15	MHWT-KHCA	40.71	0.918	40
		Tudavekar et al.	38.54	0.909	46
		Yang Li et al.	38.41	0.891	56
		Jana. Rao et al.	37.38	0.890	70
		Newson et al.	36.93	0.885	91
	30	MHWT-KHCA	38.13	0.863	59
		Tudavekar et al.	37.82	0.845	65
		Yang Li et al.	37.56	0.835	76
		Jana. Rao et al.	36.84	0.819	91
		Newson et al.	36.32	0.823	95
Girl2	15	MHWT-KHCA	39.10	0.962	37
		Tudavekar et al.	38.85	0.935	43
		Yang Li et al.	38.51	0.925	48
		Jana. Rao et al.	37.76	0.907	57
		Newson et al.	37.13	0.899	65
	30	MHWT-KHCA	37.97	0.877	55
		Tudavekar et al.	36.84	0.846	60
		Yang Li et al.	36.51	0.822	65
		Jana. Rao et al.	36.29	0.819	79
		Newson et al.	36.02	0.809	92
Surfer	15	MHWT-KHCA	38.86	0.935	48
		Tudavekar et al.	37.69	0.913	54
		Yang Li et al.	37.26	0.899	59
		Jana. Rao et al.	37.03	0.881	69
		Newson et al.	36.81	0.871	83
	30	MHWT-KHCA	36.71	0.880	61
		Tudavekar et al.	35.28	0.835	68
		Yang Li et al.	35.18	0.810	75
		Jana. Rao et al.	35.10	0.800	91
		Newson et al.	34.93	0.778	98

From the table values, it is reported that the presented MHWT-KHCA technique has accomplished maximum performance on all the applied video sequences. For instance, on the applied basketball sequence with the pixel loss of 15%, the Newson et al. method has depicted inferior results by offering a PSNR of 36.93 dB, SSIM of 0.885, and CT of 91 s. At the same time, the Jana. Rao et al. method has showcased slightly improved results by obtaining a PSNR of 37.38 dB, SSIM of 0.890, and CT of 70 s. Followed by, the Yang Li et al. method has demonstrated moderate outcome with the PSNR of 38.41 dB, SSIM of 0.891, and CT of 56 s. On continuing with, the Tudavekar et al. method has shown certainly improved outcome with the PSNR of 38.54 dB, SSIM of 0.909, and CT of 46 s. But the presented MHWT-KHCA model has exhibited superior results with the PSNR of 40.71 dB, SSIM of 0.918, and CT of 40 s.

Similarly, on the applied basketball sequence with the pixel loss of 30%, the Newson et al. model has showcased inferior outcomes by offering a PSNR of 36.32 dB, SSIM of 0.823, and CT of 95 s. Likewise, the Jana. Rao et al. technique has exhibited somewhat higher results by obtaining a PSNR of 36.84 dB, SSIM of 0.819, and CT of 91 s. Along with that, the Yang Li et al. method has demonstrated moderate outcomes with the PSNR of 37.56 dB, SSIM of 0.835, and CT of 76 s. Also, the Tudavekar et al. method has shown certainly superior outcomes with the PSNR of 37.82 dB, SSIM of 0.845, and CT of 65 s. But the proposed MHWT-KHCA model has outperformed maximum outcomes with the PSNR of 38.13 dB, SSIM of 0.863, and CT of 59 s.

Besides, on the applied Girl2 sequence with the pixel loss of 15%, the Newson et al. approach has portrayed inferior results by offering a PSNR of 37.13 dB, SSIM of 0.899, and CT of 65s. Likewise, the Jana. Rao et al. method has demonstrated slightly increased outcomes by attaining a PSNR of 37.76 dB, SSIM of 0.907, and CT of 57 s. In addition, the Yang Li et al. technique has demonstrated moderate outcomes with the PSNR of 38.51 dB, SSIM of 0.925, and CT of 48s. Besides, the Tudavekar et al. method has shown certainly improved outcomes with the PSNR of 38.85 dB, SSIM of 0.935, and CT of 43 s. However, the projected MHWT-KHCA model has showcased higher results with the PSNR of 39.10 dB, SSIM of 0.962, and CT of 37 s.

Likewise, on the applied Girl2 sequence with the pixel loss of 30%, the Newson et al. technique has outperformed inferior outcomes by offering a PSNR of 36.02 dB, SSIM of 0.809, and CT of 92 s. In line with, the Jana. Rao et al. method has showcased slightly improved results by obtaining a PSNR of 36.29 dB, SSIM of 0.819, and CT of 79s. Followed by, the Yang Li et al. method has exhibited moderate results with the PSNR of 36.51 dB, SSIM of 0.822, and CT of 65 s. Additionally, the Tudavekar et al. method has shown certainly improved outcomes with the PSNR of 36.84 dB, SSIM of 0.846, and CT of 60s. But the proposed MHWT-KHCA model has exhibited maximum outcomes with the PSNR of 37.97 dB, SSIM of 0.877, and CT of 55 s.

Simultaneously, on the applied surfer sequence with the pixel loss of 15%, the Newson et al. model has depicted inferior outcomes by offering a PSNR of 36.81 dB, SSIM of 0.871, and CT of 83s. Concurrently, the Jana. Rao et al. method has outperformed slightly higher results by obtaining a PSNR of 37.03 dB, SSIM of 0.881, and CT of 69 s. Afterward, the Yang Li et al. method has portrayed moderate results with the PSNR of 37.26dB, SSIM of 0.899, and CT of 59s. On continuing with, the Tudavekar et al. approach has shown certainly superior outcomes with the PSNR of 37.69 dB, SSIM of 0.913, and CT of 54 s. But the presented MHWT-KHCA methodology has displayed superior results with the PSNR of 38.86 dB, SSIM of 0.935, and CT of 48 s.

Finally, on the applied surfer sequence with the pixel loss of 30%, the Newson et al. method has portrayed inferior outcomes by offering a PSNR of 34.93dB, SSIM of 0.778, and CT of 98s. Simultaneously, the Jana. Rao et al. technique has showcased slightly improved results by attaining a PSNR of 35.10 dB, SSIM of 0.800, and CT of 91 s. Followed by, the Yang Li et al. approach has demonstrated moderate outcome with the PSNR of 35.18 dB, SSIM of 0.810, and CT of 75 s. Also, the Tudavekar et al. method has shown certainly improved outcomes with the PSNR of 35.28 dB, SSIM of 0.835, and CT of 68 s. But the projected MHWT-KHCA technique has demonstrated maximum outcomes with the PSNR of 36.71 dB, SSIM of 0.880, and CT of 61 s.

**Figure 7:**
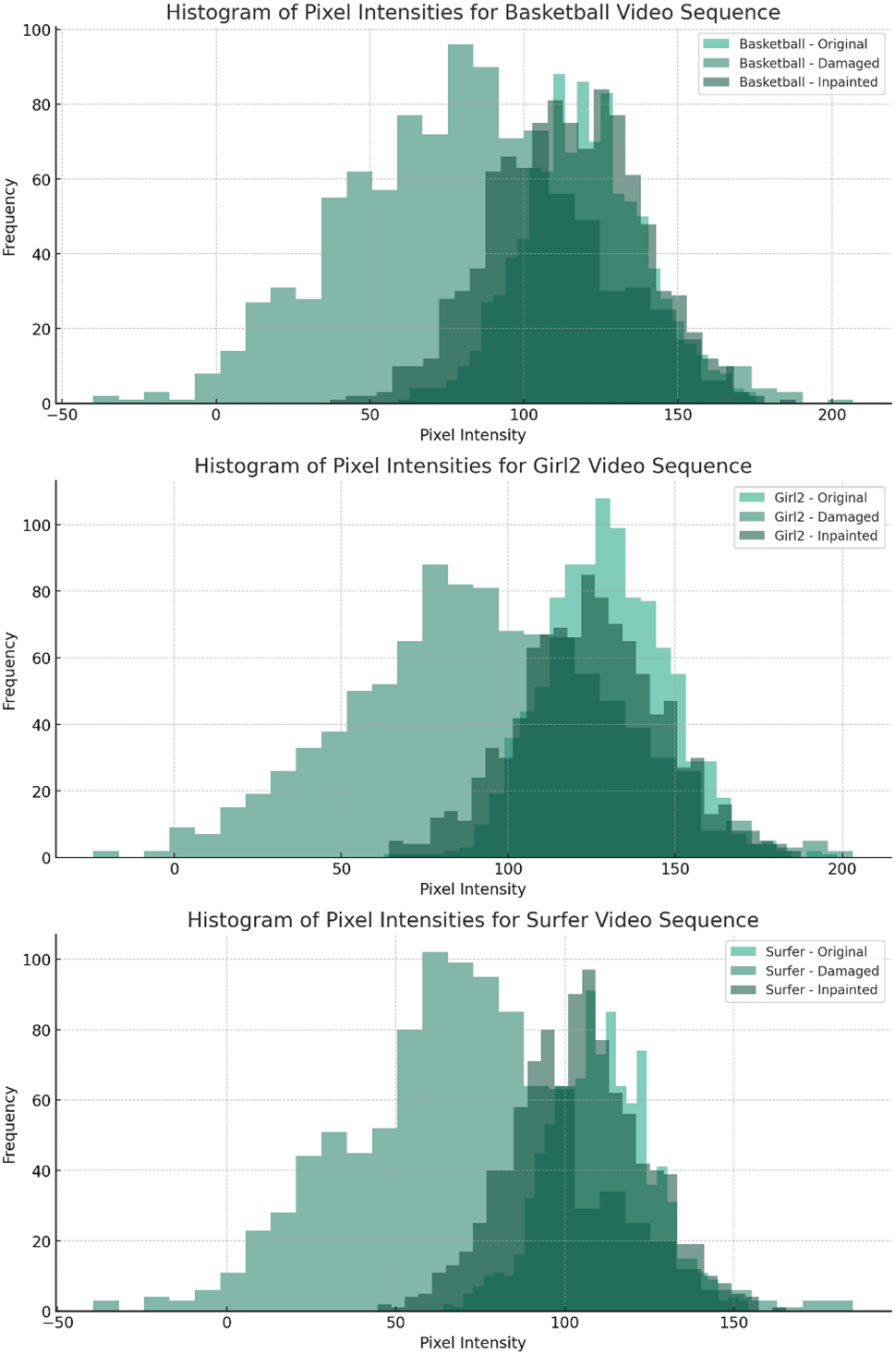
Histogram analysis (**a**) Basketball (**b**) Girl2 (**c**) Surfer.

Figure 7 illustrate the proficiency of the inpainting algorithm in restoring damaged video frames. For all three sequences, the inpainted frames' pixel intensity distributions closely align with those of the original frames, indicating that the proposed MHWT-KHCA technique effectively recovers lost or corrupted data. This alignment suggests a high degree of visual fidelity, a key outcome reinforced by the superior PSNR and SSIM values reported in the research. The close resemblance between the original and inpainted histograms is a testament to the algorithm's capability to not only repair damage but also to preserve the intrinsic features of the video content, such as texture and edge information. The improved performance demonstrated by these histograms validates the proposed work's contribution to advancing video inpainting techniques, showcasing its potential to provide high-quality, computationally efficient restoration for practical applications.

## Contributions of the research

This research introduces a novel video inpainting technique that integrates Morphological Haar Wavelet Transform (MHWT) with the Krill Herd based Criminisi algorithm (KHCA), offering significant advancements in computational efficiency and inpainting quality. The major contributions of this paper are outlined as follows:

1. We present the MHWT-KHCA algorithm that uniquely combines MHWT and KHCA. This integration utilizes the strengths of MHWT for effective multi-scale representation and edge preservation in video frames, alongside the optimization capabilities of the Krill Herd algorithm to enhance the traditional Criminisi algorithm. This integration significantly reduces the computational complexity typically associated with high-quality video inpainting processes.
2. One of the main features of the MHWT-KHCA technique is its ability to maintain high-quality inpainting while significantly reducing the time required to process videos. Traditional video inpainting methods often suffer from high computational demands, especially when dealing with high-resolution content. Our approach optimizes the search for the best matching blocks within videos, thus speeding up the inpainting process without sacrificing the output quality. This is particularly beneficial for applications requiring real-time processing, such as live video editing and broadcasting.
3. Through rigorous benchmarking using the OTB2015 dataset, our technique has demonstrated superior performance over existing methods. The proposed algorithm not only achieves higher peak signal-to-noise ratio (PSNR) and structural similarity index measure (SSIM) but also enhances the visual coherence of inpainted video frames. These improvements are critical for applications where visual quality is paramount, such as in digital restoration of archival video footage and enhancement of surveillance video clarity.
4. Our technique effectively minimizes common inpainting artifacts such as blurring and seam visibility, which are prevalent in other approaches. By utilizing the morphological properties of Haar wavelets in conjunction with the strategic patch-based optimization of the Krill Herd algorithm, our method ensures that the inpainted areas blend seamlessly with the surrounding pixels, thus maintaining the natural appearance of the video.
5. The MHWT-KHCA algorithm is not only effective for small-scale inpainting tasks but also scales efficiently to handle larger areas of missing data in videos. This versatility makes it suitable for a wide range of applications, from minor edits to significant restorations, across various genres and video formats.

## Real time/practical applications

The novel MHWT-KHCA video inpainting technique offers broad practical applications across various sectors:

- Useful for removing outdated logos, subtitles, or banners from footage, making it suitable for re-broadcasting or regional adaptation.
- Ideal for restoring damaged or deteriorated historical and cultural video archives, ensuring preservation and accessibility.
- Enhances surveillance footage clarity, aiding in investigations by inpainting obscured or missing parts due to camera malfunctions or tampering.
- Can be integrated into smartphones and cameras to allow users to automatically repair or enhance their personal video content directly on their devices.

## Conclusion

The MHWT-KHCA video inpainting technique developed in this study represents a significant advancement in the field of video restoration. This method effectively combines Morphological Haar Wavelet Transform with the Krill Herd based Criminisi algorithm to deliver a robust solution that significantly reduces computational complexity while improving inpainting quality. We demonstrated through extensive benchmarks that our technique outperforms existing methods in terms of efficiency, quality, and scalability. The potential applications of this technology span various industries, including broadcast media, digital forensics, and content creation, illustrating its practicality and wide-ranging impact. Future work will focus on integrating more advanced optimization strategies, enhancing cross-resolution capabilities, and exploring deep learning synergies to further refine and extend its applicability.

### Future prospects

The proposed MHWT-KHCA video inpainting technique offers several research opportunities, including the integration of advanced optimization algorithms to enhance efficiency and the incorporation of deep learning models to improve texture and dynamic understanding. Additionally, adapting the technique for varied video resolutions and exploring real-time applications in fields such as medical imaging and live broadcasting could significantly broaden its utility and impact across different sectors.

## Data Availability

All the data’s that support this work are included within the article.
